# The transfer of genetically engineered lymphocytes in melanoma patients: a Phase I dose escalation study

**DOI:** 10.1186/2051-1426-3-S2-P168

**Published:** 2015-11-04

**Authors:** Courtney Regan, Michael Nishimura, I Caroline Le Poole, Constantine Godellas, Patrick Stiff, Elizabeth Garrett-Mayer, Mingli Li, Keisuke Shirai, Jonathan Eby, Heather Embree, Boro Dropulic, Ann Lau Clark, Kelli Hutchens, Joseph I Clark

**Affiliations:** 1 Loyola University of Chicago Department of Medicine, Division of Hematology Oncology, Maywood, IL, USA; 2 Loyola University of Chicago, Health Sciences Division, Oncology Institute, Maywood, IL, USA; 3 Department of Surgery, Loyola University of Chicago, Maywood, IL, USA; 4 Medical University of South Carolina Department of Biostatistics & Epidemiology, Charleston, SC, USA; 5 Bluebird Bio, Cambridge, MA, USA; 6 Medical University of South Carolina, Department of Medicine, Charleston, SC, USA; 7 Lentigen Technology Inc, Gaithersburg, MD, USA; 8 Loyola University Medical Center, Department of Pathology, Maywood, IL, USA; 9 Loyola University Medical Center, Division of Hematology Oncology, Maywood, IL, USA

## Background

Genetically engineered T cells have broadened the opportunity for use of T cell immunotherapy in cancer patients. The possibility of improving T cell efficacy via introduction of additional genes potentially gives them an advantage over TIL. Studies in adoptive T cell transfer suggest that persistence is fundamental to the efficacy of this therapy. This Phase I clinical trial uses TCR TIL 1383I transduced T cells to target the tyrosinase antigen on melanoma cells. Two of the objectives in this clinical trial in stage IV melanoma patients include measuring persistence and monitoring the behavior of tumor-reactive T cells in vivo.

## Methods

Stage IV melanoma patients must have tumors that are positive for both HLA-A2 and tyrosinase on pathologic review. Peripheral blood mononuclear cells (PBMCs) are isolated from the patients and activated with anti-hCD3, rhIL2 and rhIL15. These PBMCs are transduced with lentivirus encoding the TIL 1383I TCR and expanded to treatment numbers. The transduced cells are suspended in 5% human albumin and infused over 30 minutes. The infusion is preceded by lymphodepletion with fludarabine and cyclophosphamide and followed with low dose IL-2 for one week. A modified CD34 cassette in the vector enables monitoring of the transduced T cells in the patient's PBMC post-infusion. PBMCs, complete metabolic panels, lactate dehydrogenase, and complete blood counts are collected on days 1, 3, 5, 7, 14, 25, 35 and monthly up to 3 months, and then every 1-3 months as clinically indicated. Physical exams, toxicity assessment, whole body PET/CT or CT scans, Audiology and Ophthalmologic exams are performed pretreatment and at 4 weeks post infusion and as clinically indicated every 1-3 months afterwards.

The presence of transduced T cells at each time point is measured by staining with anti-CD34 mAb and analyzed using a BD LSRFortessa flow cytometer.

## Conclusion

This study is open to accrual at Loyola University Medical Center and is in the process of expanding to other institutions. We have successfully completed treatment in 3 of 55 screened patients, with goal accrual of 24 patients. Results to date confirm that infused genetically engineered TIL 1383I TCR transduced T cells are detectable more than 6 months after infusion and demonstrate activity. We plan to move forward with a Phase II study.
